# Synthesis and Biological Evaluation of Novel 1-Alkyl-tryptophan Analogs as Potential Antitumor Agents

**DOI:** 10.3390/molecules14125339

**Published:** 2009-12-18

**Authors:** Ting Sun, Zhao-Long Li, Hua Tian, Shih-Chen Wang, Jiong Cai

**Affiliations:** 1 Department of Nuclear Medicine, Peking Union Medical College & Chinese Academy of Medical Sciences, Peking Union Medical College Hospital, Beijing 100730, China; E-Mails: beibeisun2008@163.com (T.S.); jiongcai@sina.com (J.C.); 2 Department of Chemistry, Tsinghua University, Beijing 100084, China; E-Mail: zhlli@tsinghua.edu.cn (Z.-L.L.); 3 Department of Pathology, Shanghai Jiao Tong University School of Medicine, Shanghai 200025, China; E-Mail: huatian722@sohu.com (H.T.)

**Keywords:** tryptophan analogs, cytotoxicity, MTT

## Abstract

To seek novel antitumor agents, we designed and synthesized new 1-tryptophan analogs based on tryptophan catabolism. 1-Alkyltryptophan analogues including 1-ethyl-tryptophan (1-ET), 1-propyltryptophan (1-PT), 1-isopropyltryptophan (1-isoPT) and 1-butyltryptophan (1-BT) were synthesized from tryptophan. We examined whether those compounds had the antiproliferative effects on SGC7901 and HeLa cells line by using MTT assay *in vitro*, respectively. Compared to tryptophan, all targeted compounds efficiently inhibited proliferation of two cancer cell lines at 2 mmol/L for 48 hours. Among these tryptophan analogs, 1-BT showed the most powerful cytotoxicity against SGC7901 and HeLa cells at 1 mmol/L and 2 mmol/L concentration. These data suggest that some specific tryptophan analogs could be developed as potential anti-neoplastic agents.

## 1. Introduction

Tumor cells have the property of unlimited malignant growth. The development of tumors is highly dependent on the nutrients that cells receive through the blood. Like all other cells, tumor cells require amino acids for proliferation [1]. Most tumor cells have the ability to concentrate amino acids more effectively than normal cells [2,3]. Tryptophan is an indispensable amino acid required for biosynthesis of proteins in the normal tissues, which plays many crucial roles in cell proliferation [4,5]. A recent study reveals that tryptophan plays an important role in the proliferation of tumor cells [6]. Studies show that there are active L-type amino acid transporters in tumor cells for uptaking more essential amino acids [7,8].

Besides the high rate of tryptophan incorporation in proteins, tumor cells may show active tryptophan degradation along the so-called kynurenine pathway [9,10]. The fate of tryptophan analogs in tumor cells is catabolism or incorporation in proteins [11]. Based on these results, we have hypothesized that redundant tryptophan analogs may have effect on viability and proliferation in tumor cells. It is not reported the relationship between the alkyl groups at the N-H bond of the indole ring and biological activity on tumor cells. We designed some 1-alkyltryptophan analogs and observed their antitumor activity *in vitro*, compared with 1-methyl-dl-tryptophan (1-MT). We also present here a new synthetic method of tryptophan analogs from l-tryptophan.

## 2. Results and Discussion

### 2.1. Chemistry

Since the indole ring of tryptophan was hydrolyzed into an aromatic amine in the tryptophan-kynurenine pathway [12], we modified indole ring to form 1-alkyltryptophan whose metabolites were different from kynurenine. Moreover alkylation at the site of active hydrogen in the indole ring of tryptophan was considered feasible [13]. Unlike the common biosynthesis of natural amino acids and the complicated asymmetric synthesis of unnatural amino acids [14], we used simple chemical reactions to synthesize 1-ethyltryptophan (1-ET), 1-propyltryptophan (1-PT), 1-isopropyltryptophan (1-isoPT) and 1butyl-tryptophan (1-BT), starting from tryptophan. 

**Scheme 1:**
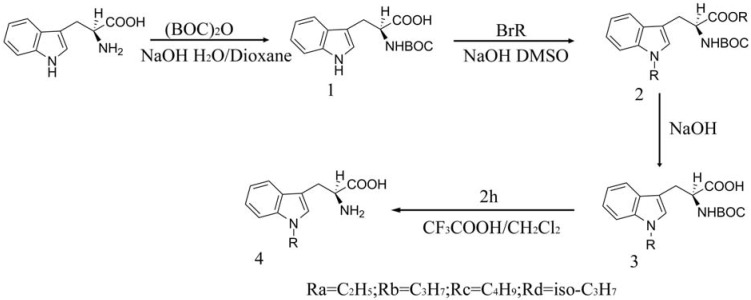
Synthesis of 1-alkyl-Trp from Trp.

Tryptophan had three active groups including the indole N-H, carboxyl and amino groups, of which the indole N-H was the least active group under alkaline conditions. The amino group of tryptophan was protected with di-*tert*-butyl dicarbonate to give Boc-*N*-Trp [15]. The carboxyl protecting and indole alkylating reactions were carried out at one step using three equivalents of alkyl bromide in the presence of twofold alkali and dimethyl sulphoxide. Finally, we got the 1-alkylTrp compounds after carboxyl and amino group deprotection reactions. (Scheme 1) These analogs were all obtained under similar condition, but the yield of 1-isoPT (32%) was lower than that of the other compounds (47–60%) due to the steric effects of the alkyl group.

The optical rotation of alpha amino acid is changed in a strong acid or alkali [16]. In our study, we used strong acid and alkali reactions, which resulted in the change of the optical rotation of l-tryptophan. Optical rotation measurements suggested these products were racemic mixtures. (Table 1) Due to conflicting results of which isomer would be better suited as an promising therapeutic agent, most studies to date have employed the racemic mixture 1-MT (that is, a mixture of the d and l isomers of 1-MT) [17]. Recently, Inaba, T. *et al.* reported 1-methyl-dl-tryptophan abrogated the progress of advanced ovarian cancer in mice [18]. Thereby, we synthesized these tryptophan analogs as the racemic mixture without chiral separation in our study.

### 2.2. Biological activity evaluation

**Figure 1:**
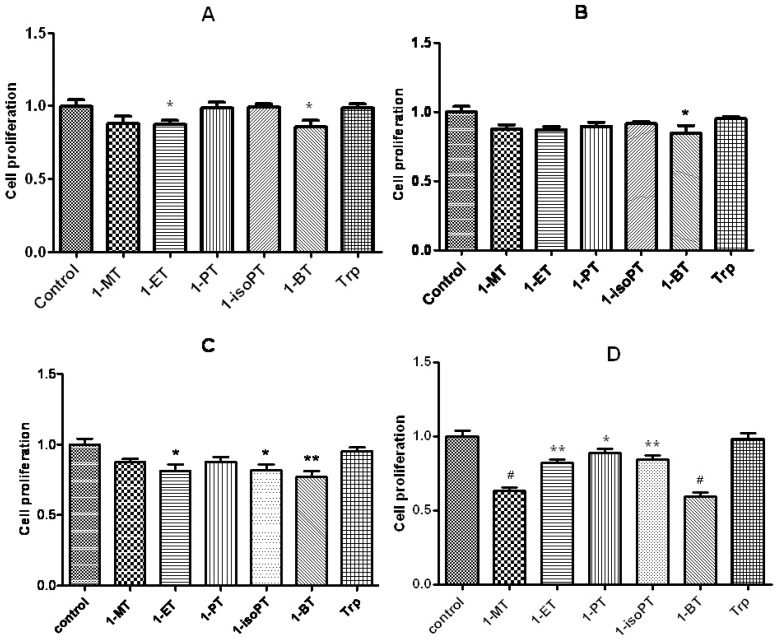
The effects of various concentrations of target compounds on SGC7901 cell proliferation. Cells were treated with various concentrations of the compounds (A) 0.1 mmol/L (B) 0.5 mmol/L (C) 1 mmol/L (D) 2 mmol/L for 48 hours and the relative cell viability was determined by MTT. The values are expressed as mean ± SD of three determinations.* *p* < 0.05, compared with Trp. ** *p* < 0.01, compared with Trp. ^#^ *P* < 0.01, compared with 1-ET.

**Figure 2:**
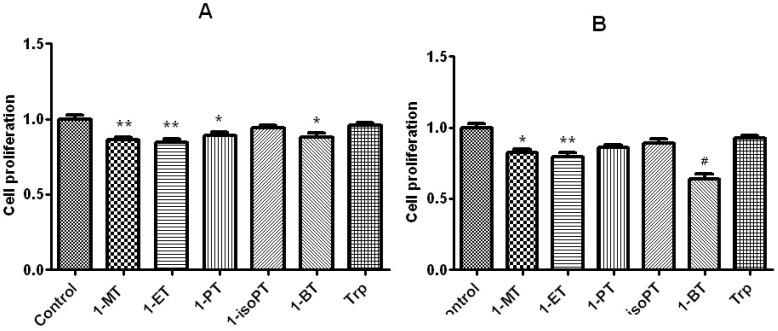

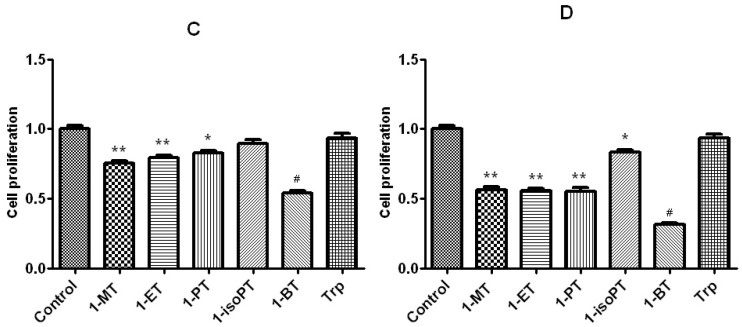
The effect of various concentrations of target compounds on HeLa cell proliferation. Cells were treated with various concentrations of compounds (A) 0.1 mmol/L (B) 0.5 mmol/L (C) 1 mmol/L (D) 2 mmol/L for 48 hours and the relative cell viability was determined by MTT. The values are expressed as mean ± SD of three determinations. * *p* < 0.05, compared with Trp. ** *p* < 0.01, compared with Trp. # *p* < 0.01, compared with any other group.

**Figure 3:**
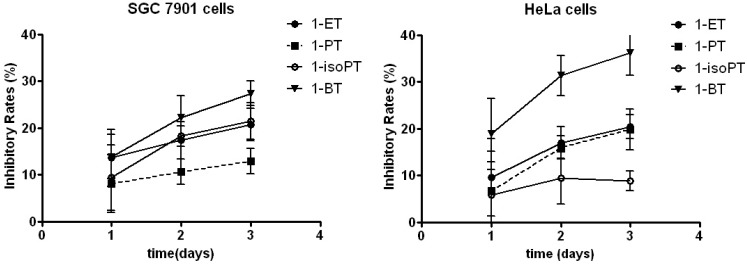
The effect of 1 mmol/L of target compounds on SGC7901and Hela cells growth. Cells were treated with 1 mmol/L compounds for 1, 2 and 3 days and the relative cell viability was determined by MTT. The values are expressed as mean ± SD of three determinations.* *p* < 0.05, compared with control. ** *p* < 0.01, compared with control.

Gastroenteric tumor and cervical carcinoma are the most common cancers in China, so we studied the anti-proliferative effects of the 1-alkyltryptophanes on SGC7901 cells and HeLa cells. Our data suggested that these analogues inhibited the proliferation of the two cancer cell lines in a dose-dependent manner, as shown in Figure 1B,C,D and Figure 2B,C,D. All compounds had no obvious activity at 0.01 mmol/L, but showed antiproliferative effects at 2 mmol/L. Only 1-BT at a concentration of 0.1 mmol/L inhibited proliferation of SGC7901 cells after 48 hours of treatment (Figure 1A). All analogues suppressed HeLa cells proliferation at 0.1 mmol/L, but 1-isoPT. 1-BT was more potent than others (Figure 1D and Figure 2D), which suggested that 1-BT could be a potential antitumor compound. Next, we investigated the time-proliferation curves with each analogue (1 mmol/L). As shown in Figure 3, the activities of these analogs were in a time-dependent manner. Compared with SGC7901 cells, their antiproliferative effects were more significant on HeLa cells at the same concentration, which suggested HeLa cells were more sensitive to 1-alkly-tryptophan analogs than SGC7901 cells.

Obviously, the biochemcial mechanism of enhanced cytotoxicity on two cancer cell lines was highly dependent on the structure of the 1-indole side chains. 1-isoPT had the largest sterically hindered group among these analogs, whereas it had the lowest cytotoxicity at any concentration. At all given dosis, 1-BT had the most efficient antitumor activity among these analogs. These results hinted that the cytotoxicity was closely related to the length of carbon chain attached at the indole *N*. It could be assumed that length of carbon chain was important for cytotoxicity.

The detailed mechanisms accounting for the antitumor activity of tryptophan analogs is still unclear, so we propose a number of possible reasons based on the uptake and metabolism of amino acids. Tumor cells have the properties of an infinite lifespan and vigorously grow without limit, so they obtain adequate amounts of amino acids with increased amino acid transporters [1,19,20]. Tryptophan analogs, serving as an essential amino acid, may mistakenly be transported into tumor cells [21]. Whether these analogs may be metabolized or incorporated into protein, they could change tumor cell division and growth. The underlying mechanisms would be related to space structure or steric effect of these analogs themselves, which could occupy some transporters resulting in the decreased uptake of other amino acids, or change the biological activity of protein and enzyme, or produce poisonous essential metabolites.

## 3. Experimental

### 3.1. General

Melting points were determined on a Yanaco melting point apparatus (MP-S3 model) or Round Science RFS-10. ^1^H- and ^13^C-NMR spectra were recorded on a JEOL AL-300MHz NMR spectrometer at ambient temperature. The 1-methyl-DL-tryptophan (1-MT) was obtained from Sigma (St Louis, MO, USA). The L-tryptophan (Trp) was obtained from Sinopharm Chemical Reagent Beijing Co., Ltd. MTT was obtained from Sigma (St Louis, MO, USA). 1-ET, 1-PT, 1-BT and 1-isoPT were dissolved in PBS and sterilized by passage through a 0.22-mm Millipore filter into a sterile multidose vial.

### 3.2. Synthesis of tryptophan anologs

*N-Boc-l-tryptophan* (**1**). Di-tert-butyl dicarbonate (10.09 g, 50 mmol) and 1 M NaOH (50 mL) were added to a solution of L-tryptophan (10.2 g, 50 mmol) in 1:1 water–dioxane (200 mL). The mixture was stirred at room temperature for 24 h, then the pH was adjusted to 2.4 by adding aqueous HCl, and the mixture was extracted with EtOAc (2 × 150 mL). The organic phase was evaporated to dryness to give *N*-Boc-l-tryptophan (10.5 g, 69% yield) as a white solid. m.p.: 136–138 °C; ^1^H-NMR (CDCl_3_): 8.13 (br, 1H, Ind-NH), 7.61 (d, 1H, *J* = 7.6 Hz, Ind-7-H), 7.37 (d, 1H, *J* = 8.1 Hz, Ind-4-H), 7.15–7.11 (m,2H, Ind-5,6-H), 7.03 (s, 1H, Ind-2-H), 5.06 (br, 1H, NHBoc), 4.68–4.64 (m, 1H, C*H), 3.37–3.31 (m, 2H,CH_2_) 1.43 (s, 9H, CH_3_). 

*2-(tert-Butoxycarbonylamino)-3-(1-ethyl-1H-indol-3-yl)propanoate* (**2a**). *N*-Boc-l-tryptophan (3.04 g, 10 mmol) and NaOH (0.8 g, 20 mmol) were dissolved in dried DMSO (20 mL). The mixture was stirred for 2 h at 40 °C, then bromoethane (3.24 g, 30 mmol) was added. After stirring another 4 h, H_2_O (120 mL) was added and the mixture was extracted with EtOAc (2 × 30 mL). Then the organic phase was collected, dried over anhydrous Na_2_SO_4_, and evaporated to give the crude compound (3.2 g). The crude product was purified by column chromatography on silica gel using petroleum ether-EtOAc (10:1) as eluant to obtain pure **2a** (2.16 g, 6 mmol, 60% yield) as a white solid. m.p.: 96-98 °C; ^1^H- NMR (CDCl_3_): δ 7.55 (d, 1H, *J* = 7.92 Hz), 7.30 (d, 1H, *J* = 7.89 Hz), 7.07-7.24 (m, 2H), 6.92 (s, 1H), 5.07 (br, 1H), 4.62 (br, 1H), 4.11 (m, 4H), 3.28 (br, 2H), 1.43 (m, 12H), 1.20 (t, 3H); ^13^C-NMR (CDCl_3_): δ (ppm) 172.5, 155.4, 136.0, 128.5, 125.8, 121.7, 119.1, 109.4, 108.9, 78.1, 61.4, 54.4, 40.9, 28.5, 28.1, 15.6, 14.2.

*2-(tert-Butoxycarbonylamino)-3-(1-propyl-1H-indol-3-yl)propanoate* (**2b**). The title compound was synthesized from *N*-Boc-l-tryptophan (3.04 g, 10 mmol) and NaOH (0.8 g, 20 mmol) and 1-bromopropane (3.69 g, 30 mmol), as described above for **2a**. Yield: 2.23 g, 5.7 mmol 57.5%; ^1^H-NMR (CDCl_3_): δ 7.57 (d, 1H, *J* = 7.89 Hz), 7.31 (d, 1H, *J* = 7.89 Hz), 7.20 (t, 1H, *J* = 7.55 Hz), 7.10 (t, 1H, *J* = 7.38 Hz), 6.92 (s, 1H), 5.10 (d, 1H, *J* = 7.23 Hz), 4.65 (m, 1H), 4.03 (m, 4H), 3.29 (br, 2H), 1.85 (m, 2H), 1.60 (m, 2H), 1.46 (s, 9H), 0.89 (m, 6H); ^13^C-NMR (CDCl_3_): δ (ppm) 172.5, 155.3, 136.3, 128.4, 126.6, 121.6, 119.0, 109.5, 108.6, 79.7, 66.9, 54.4, 48.0, 28.4, 28.1, 23.5, 21.9, 11.6, 10.3.

*2-(tert-Butoxycarbonylamino)-3-(1-butyl-1H-indol-3-yl)propanoate* (**2c**). The syntheses of **2c**, starting from *N*-Boc-l-tryptophan (3.04 g, 10 mmol) and NaOH (0.8 g, 20 mmol) and 1-bromobutane (4.11 g, 30 mmol) at 60 °C, was performed as described for **2a**. Yield: 2.05 g, 4.9 mmol, 49%; ^1^H-NMR (CDCl_3_): δ 7.57 (d, 1H, *J* = 7.89 Hz), 7.31 (d, 1H, *J* = 8.22 Hz), 7.21 (t, 1H, *J* = 7.56 Hz), 7.10 (t, 1H, *J* = 7.38 Hz), 6.91 (s, 1H), 5.11 (br, 1H), 4.64 (m, 1H), 4.07 (m, 4H), 3.29 (d, 2H, *J* = 4.80 Hz), 1.79 (m, 2H), 1.54 (m, 2H), 1.46 (s, 9H), 1.23-1.36 (m, 4H), 0.86-0.97 (m, 6H); ^13^C-NMR (CDCl_3_): δ (ppm) 172.5, 155.3, 136.2, 128.4, 126.5, 121.6, 119.0, 109.4, 108.7, 79.6, 65.1, 54.4, 45.9, 32.3, 30.5, 28.4, 28.1, 20.2, 19.1, 13.7.

*2-(tert-Butoxycarbonylamino)-3-(1-isopropyl-1H-indol-3-yl)propanoate* (**2d**). The syntheses of **2d**, starting from *N*-Boc-l-tryptophan (3.04 g, 10 mmol) and NaOH (1.0 g, 25 mmol) and 2-bromopropane (3.69 g, 30 mmol) at 80 °C, was performed as described for **2a**. Yield: 1.26 g, 3.2 mmol 32%; ^1^H-NMR (CDCl_3_): δ 7.58 (d, 1H, *J* = 7.89 Hz), 7.30 (d, 1H, *J* = 8.22 Hz), 7.18 (t, 1H, *J* = 7.56 Hz), 7.08 (t, 1H, *J* = 7.56 Hz), 5.22 (d, 1H, *J* = 7.89 Hz), 4.96 (m, 1H), 4.52-4.66 (m, 2H), 3.27 (m, 2H), 1.44 (m, 15H), 1.14 (m, 6H); ^13^C-NMR (CDCl_3_): δ (ppm) 171.5, 155.0, 135.4, 128.1, 121.9, 121.1, 118.8, 118.7, 109.1, 108.8, 79.1, 68.5, 54.3, 46.5, 28.0, 27.8, 21.4, 21.3.

*1-Ethyltryptophan* (**4a**). 2-(tert-Butoxycarbonylamino)-3-(1-ethyl-1H-indol-3-yl)propanoate (**2a**, 2.16 g, 6 mmol) was stirred with 1M NaOH (9 mL 1.5 equiv) in DMSO (20 mL) at room temperature for 30 min. Excess H_2_O was added to the reaction and washed with EtOAc. The aqueous adjusted pH to 2.4 and extracted threes times with EtOAc. The combined organic extracts were concentrated under reduced pressure and got the crude product. The crude product was dissolved in CH_2_Cl_2_-CF_3_COOH (4:1, 20 mL) and stirred for 2 h. The solvent was evaporated and the crude product was dissolved in water (10 mL), then filtered after adjusting the pH to 7.0 to give pure 1-ethyltryptophan (**4a**) as a white solid (0.93 g, 4 mmol 67% yield); m.p.: 130-132 °C; ^1^H-NMR (DMSO-*d_6_*): δ 8.31 (br, 2H, *J* = 7.89 Hz), 7.58 (d, 1H, *J* = 7.89 Hz), 7.45 (d, 1H, *J* = 8.25 Hz), 7.26 (s, 1H), 7.15 (t, 1H, *J* = 7.38 Hz), 7.04 (t, 1H, *J* = 7.38 Hz), 4.11-4.20 (m, 3H), 3.27 (br, 2H), 1.35(t, 3H); ^13^C-NMR (DMSO-*d6*): δ (ppm) 170.9, 135.8, 127.6, 127.5, 121.3, 118.7, 118.6, 109.8, 106.3, 52.7, 40.3, 26.1, 15.3.

*1-Propyltryptophan* (**4b**). Compound **4b** was synthesized from **2b** following the procedure described for the preparation of **4a**. It is a white solid, m.p.: 158-161 °C; ^1^H-NMR (D_2_O): δ 7.60 (d, 1H, *J* = 7.89 Hz), 7.43 (d, 1H, *J* = 8.25 Hz), 7.10-7.22 (m, 3H), 4.28 (t, 1H, *J* = 5.85 Hz), 4.02 (t, 2H, *J* = 6.87 Hz), 3.36 (m, 2H), 1.73 (m, 2H), 0.74(t, 3H); ^13^C-NMR (\D_2_O): δ (ppm) 172.0, 136.3, 128.9, 127.1, 122.0, 119.4, 118.5, 110.5, 105.3, 53.5, 47.5, 25.8, 28.1, 23.0, 10.7.

*1-Butyltryptophan* (**4c**). Compound **4c** was synthesized from **2c** in 65% yield by the described procedure for the preparation of **4a**. It is a white solid, m.p.: 138-141 °C; ^1^H-NMR (D_2_O): δ 7.36 (d, 1H, *J* = 7.89 Hz), 7.22 (d, 1H, *J* = 8.25 Hz), 6.95-7.01 (m, 2H), 6.87 (t, 1H, *J* = 7.53 Hz), 4.08 (t, 1H, *J* = 6.02 Hz), 3.85 (t, 2H), 3.15 (br, 2H), 1.46 (m, 2H), 0.89 (m, 2H), 0.55(t, 3H); ^13^C-NMR (D_2_O): δ (ppm) 171.8, 136.2, 128.8, 127.0, 121.9, 119.4, 118.4, 110.4, 105.1, 53.3, 45.5, 31.6, 25.6, 19.4, 12.9. 

*1-Isopropyltryptophan* (**4d**). Compound **4d** was synthesized from **2d** in 65% yield by the described procedure for the preparation of **4a**. It is a white solid, m.p.: 169-171 °C; ^1^H-NMR (D_2_O): δ 7.59 (d, 1H, *J* = 7.89 Hz), 7.42 (d, 1H, *J* = 7.92 Hz), 7.23 (s, 1H), 7.01-7.22 (m, 2H), 4.58 (m, 1H), 4.25 (t, 1H, *J* = 6.18 Hz), 3.34 (m, 2H), 1.37 (d, 2H); ^13^C-NMR (D_2_O): δ (ppm) 171.9, 135.7, 127.1, 124.7, 121.8, 119.5, 118.5, 110.4, 105.7, 53.4, 47.2, 25.8, 21.7.

### 3.3. Sample preparation and optical rotation measurements

A Perkin-Elmer 343 polarimeter was used. All measurements were carried out at 20 °C using the sodium D line (589 nm). The cell path length was 10 cm. Water was purified with a Milli-Q water system (Organex) supplied by Millipore (resistivity > 18 MΩ·cm). The samples were prepared by weighing the appropriate amount of 1-alkyltryptophan analog, using as solvent the previously prepared water. All samples were left to stand for one day before measuring the optical rotation. Each sample was prepared, and measurements were made at least twice. The specific rotation or optical activity is expressed as:

$${\left[\alpha \right]}_{D}^{T}=\frac{100\alpha }{l\times c}$$

In this equation, l is the path length in decimeters and c is the concentration in g/100 mL, for a sample at a temperature T (given in degrees Celsius) and wavelength D (in nanometers). 

### 3.4. Cell lines and culture conditions

Human SGC 7901 (gastric cancer) and HeLa (cervical carcinoma) cell lines were obtained from the Cell Bank of Shanghai Institute of Cell Biology of the Chinese Academy of Sciences. Cells were cultured in RPMI 1640 medium (Sigma, St Louis, Missouri, USA), supplemented with 10% fetal calf serum and containing penicillin (100 U/mL) and streptomycin (100 μg/mL) at 37 °C in a humidified atmosphere containing 5% CO_2_.

### 3.5. MTT assay

Briefly, all cells in the exponential growth phase were plated in a 96-well plate at 2 × 10^3^ cells/well, and incubated at 37 °C for 24 hours. The cells were then incubated with tryptophan analogues. After a further time in culture, 10 μL of MTT (3-[4, 5-dimethylthiazol-2-yl]-2,5-diphenyltetrazolium bromide) (5mg/mL, Sigma) was added to each well and incubated again for four hours at 37 °C. MTT was then removed and 150 μL of DMSO was added to each well. The absorbance at 490 nm was determined using an ELISA reader (American Research Company, USA). All manipulations were repeated and performed in quintuple.

### 3.6. Statistical analysis

All results are presented as mean ± S.D. SPSS 13.0 statistical package (SPSS, Inc., Chicago, IL, USA) was used to analyze the experimental data. Multiple comparisons were done with one-way analysis of variance (ANOVA). Two group comparisons were performed with a *t*-test. Statistical differences with *p* values less than 0.05 were considered to be significant.

## 4. Conclusions

In summary, we used simple chemical reactions to synthesize 1-alkyltryptophan derivatives 1-ET, 1-PT, 1-BT and 1-isoPT, which inhibited the proliferation of SGC7901 and HeLa cells at 2 mmol/L concentration. Among these analogs, 1-BT was the most competent one and could be a potential antitumor compound. The mechanism of the observed antitumor activity needs further investigation.

## Figures and Tables

**Table 1:** Concentration (C, in g/100 mL), specific rotation [α]_D_^T^ for 1-alkyl-tryptophan analogs at 589 nm at temperature *T* and concentration *c* (in g/100 mL; at 20 °C).

Compounds	C	[α]_D_^T^ (T=20 °C; *c*=1)		
	g/100mL	in H_2_O	in 1 M HCl	in 1 M NaOH
1-ET	0.19	0	+5.8	+14
1-PT	0.17	-2.5	+8.8	+17.7
1-isoPT	0.14	-2.8	+5.7	+10.0
1-BT	0.14	0	+10.0	+18.7
